# NLRP3 Inflammasome and Caspase-1/11 Pathway Orchestrate Different Outcomes in the Host Protection Against *Trypanosoma cruzi* Acute Infection

**DOI:** 10.3389/fimmu.2018.00913

**Published:** 2018-05-03

**Authors:** Augusto F. Paroli, Patricia V. Gonzalez, Cintia Díaz-Luján, Luisina I. Onofrio, Alfredo Arocena, Roxana C. Cano, Eugenio A. Carrera-Silva, Susana Gea

**Affiliations:** ^1^Centro de Investigaciones en Bioquímica Clínica e Inmunología (CIBICI – CONICET), Departamento de Bioquímica Clínica, Facultad de Ciencias Químicas, Universidad Nacional de Córdoba, Córdoba, Argentina; ^2^Instituto de Biología Celular, Facultad de Medicina, Universidad Nacional de Córdoba, Córdoba, Argentina; ^3^Instituto de Medicina Experimental (IMEX-CONICET), Academia Nacional de Medicina, Buenos Aires, Argentina

**Keywords:** *Trypanosoma cruzi*, Chagas disease, NLRP3, caspase-1/11, inflammasome, macrophages, liver

## Abstract

Infection with protozoan parasite *Trypanosoma cruzi* results in activation of nucleotide-binding domain and leucine-rich repeat containing receptors (NLRs). NLR activation leads to inflammasome formation, the activation of caspase-1, and the subsequent cleavage of IL-1β and IL-18. Considering that inflammasome activation and IL-1β induction by macrophages are key players for an appropriate T cell response, we investigated the relevance of NLR pyrin domain-containing 3 (NLRP3) and caspase-1/11 to elucidate their roles in the induction of different T cell phenotypes and the relationship with parasite load and hepatic inflammation during *T. cruzi-*Tulahuen strain acute infection. We demonstrated that infected *nlrp3−/−* and C57BL/6 wild type (WT) mice exhibited similar parasitemia and survival, although the parasite load was higher in the livers of *nlrp3−/−* mice than in those of WT mice. Increased levels of transaminases and pro-inflammatory cytokines were found in the plasma of WT and *nlrp3−/−* mice indicating that NLRP3 is dispensable to control the parasitemia but it is required for a better clearance of parasites in the liver. Importantly, we have found that NLRP3 and caspase-1/11-deficient mice differentially modulate T helper (Th1, Th2, and Th17) and cytotoxic T lymphocyte phenotypes. Strikingly, *caspase-1/11−/−* mice showed the most dramatic reduction in the number of IFN-γ- and IL-17-producing CD4+ and CD8+ T cells associated with higher parasitemia and lower survival. Additionally, *caspase-1/11−/−* mice demonstrated significantly reduced liver inflammation with the lowest alanine aminotransferase (ALT) levels but the highest hepatic parasitic load. These results unequivocally demonstrate that caspase-1/11 pathway plays an important role in the induction of liver adaptive immunity against this parasite infection as well as in hepatic inflammation.

## Introduction

Chagas disease is a neglected disease caused by the obligate intracellular protozoan parasite *Trypanosoma cruzi* that affects millions of people in Latin America. As a result of global warming and human migration, this disease is now a global public health issue. This has led to an increased risk of transmission of the infection, mainly through blood transfusion and organ transplantation (1). Chagas disease is characterized by two distinct phases, an acute phase involving a number of parasites detected in the bloodstream as well as in host tissues, and after parasitemia is controlled, a chronic phase established and represented by subpatent parasitism and positive serology. Approximately 70% of the patients in this phase present with an indeterminate form, while 30% will develop clinically different cardiac, digestive, or cardio-digestive forms of the disease decades after the infection (2). Based on epidemiological findings as well as on animal models, it has been proposed that this heterogeneous clinical response is linked to host genetic background and the extremely high genetic diversity of *T. cruzi* (3, 4). Parasite strains exhibit different organ tropism during acute and chronic infection (5). In this study, we used *T. cruzi*-Tulahuen strain that mainly infects liver and spleen and induces liver pathology and splenomegaly during acute phase (6). Besides, this parasite strain also infects heart contributing to the development of chagasic cardiomyopathy during the chronic phase. Our research group previously demonstrated that during the acute infection with the Tulahuen strain, the liver of C57BL/6 mice displayed numerous cellular infiltrates with predominance of macrophages, granulocytes, and T lymphocytes associated with high expression of TLR9 on these cells and persistent levels of pro-inflammatory cytokines. These results were correlated with higher transaminase activity (7). Even though macrophages, both classically and alternatively activated, represent immune effectors, and they are also potential reservoirs for *T. cruzi* (8, 9).

The simultaneous occurrence of different T helper (Th) cells subsets and the outcome of *T. cruzi* infection could be defined as a battle between beneficial Th1 response that fights against intracellular parasites and Th2 response that promotes parasite’s replication (7, 10). An important protective role of Th17 cells in the control of parasitemia and survival of *T. cruzi*-infected mice was recently reported (11). Protective immune responses against this parasite are based on the release of IL-12 by infected macrophages, which induce IFN-γ production from natural killer and T cells (12). IFN-γ activates nitric oxide synthase and NADPH oxidase expression and the production of reactive nitrogen intermediates and reactive oxygen species (ROS) in macrophages, which are critical for their trypanocidal activity (13, 14). Tumor necrosis factor (TNF) also plays a role in amplifying microbicidal mechanisms of IFN-γ-activated macrophages and thus contributes to host protection (15). IFN-γ is also important for orchestrating the ongoing adaptive immunity, contributing to differentiation of CD4+ Th1 and CD8+ T cells, required for the control of the parasite multiplication occurring during the acute infection. Among the various CD8+ T cell subsets, Tc1 were shown to play a major role in the fight against *T. cruzi* (16). The hallmark of this subset is the production of IFN-γ and TNF. In addition to killing and releasing cytokines, recent studies have ascribed a novel regulatory role to CD8+ T cells (17, 18). Regulatory CD8+ T cells represent a transient state of effector CD8+ T cells which promote the production of the immunosuppressive IL-10 cytokine to counteract inflammation (19).

The innate immune system utilizes different sets of germline-encoded receptors to detect *T. cruzi* infection. These include the toll-like receptor (TLR) family members that are expressed at the plasma membrane and in endosomal compartments (20). Additionally, the intracellular Nod-like receptor (NLR) family comprises several members capable of assembling inflammasomes once they get activated. The different types of inflammasomes are distinguished by their NLRs, adaptors, and stimuli specificities. The NLR pyrin domain-containing 3 (NLRP3) inflammasome, which is possibly the most extensively studied platform, is activated by pathogen-associated molecular patterns and damage-associated molecular patterns. The central adaptor molecule, which is required for the assembly of the NLRP3 inflammasome, is the apoptosis-associated speck-like protein containing a caspase recruitment domain (ASC). This pyrin-containing protein recruits pro-caspase-1 and allows its interaction with NLRP3 (21). The inflammasomes promote the activation of caspase-1 whose pro-inflammatory activity consists in catalyzing the maturation of pro-IL-1β and pro-IL-18 into its bioactive forms (22). IL-1β induces an increase in the number of activated CD4 and CD8 cells and augments the differentiation of the antigen-triggered T cells (23). IL-1β was also shown to promote IL-17 production highlighting the tight link between them (24). In concordance with this, CD4+ and CD8+ T cells produce IFN-γ that further activate phagocytic cells to promote parasite killing (25). Additionally, it was recently demonstrated that IL-1β is relevant for appropriate antigen presentation in host defense (26, 27).

Previously, it was reported that NLRP3-deficient mice exhibited higher parasitemia suggesting more susceptibility to infection with the *T. cruzi*-Y strain, whereas another group described that NLRP3 participation was not crucial for the resistance to parasite using bone marrow macrophages infected with the SilvioX10/4 strain (28–30). The aim of this work was to investigate the role of both NLRP3 and caspase-1/11 in the induction of the adaptive immunity, the different T cell subsets emerged, and their relationship with parasite load and hepatic inflammation during *T. cruzi-*Tulahuen strain acute infection.

Our results demonstrate for the first time that NLRP3 is dispensable for controlling parasitemia, though it is relevant for killing of parasites in the liver. Our data, also demonstrate that lacking caspase-1/11 pathways profoundly alters the hepatic T helper and CD8+ T cell phenotypes during *T. cruzi* infection. Moreover, caspase-1/11 deficiency skews the immune response to a dominant Th2 cytokine profile indicating that caspase-1/11 is important for both the anti-parasite T cell immunity and liver inflammation.

## Materials and Methods

### Ethic Statement

All animal experiments were approved by and conducted in accordance with guidelines of the committee for Animal Care and Use of the Facultad de Ciencias Químicas, Universidad Nacional de Córdoba (Approval Number HCD 388/11) in strict accordance with the recommendation of the Guide to the Care and Use of Experimental Animals published by the Canadian Council on Animal Care (OLAW Assurance number A5802-01).

### Mice, Infection, Parasitemia, and Survival

C57BL/6 (B6) mice indicated as wild type (WT) along the manuscript, were purchased from National University of La Plata (Bs. As., Argentina). *Nlrp3−/−* and *casp-1/11−/−* mice were obtained from Jackson Laboratory (Bar Harbor, ME, USA). Animals were maintained at the Animal Resource Facility of the CIBICI-CONICET (NIH-USA assurance number A5802-01) at 22 ± 2°C with a 12 h light–dark cycle, with food and water *ad libitum*. Six- to eight-week-old male mice were intraperitoneally injected with 10^3^ blood trypomastigotes-Tulahuen strain. Blood and livers were collected at 14 and 21 days post infection (dpi). Survival of each mouse was followed every day. Parasitemia was measured as previously described (31). Uninfected mice of each strain were used as controls. Parasites were maintained by serial passages from mouse-to-mouse each 14 days in B6 mice.

### Quantitative PCR

*Trypanosoma cruzi* was quantified by detection of parasite’s satDNA amplified in triplicate using TaqMan MGB probes and the TaqMan Universal PCR Master Mix (Applied Biosystems) on a StepOne Plus cycler (Applied Biosystems) as previously described (32). Parasite DNA and gene expression by real-time PCR was quantified by the comparative threshold cycle (CT) method (RQ = 2^−ΔΔT^), using normalization with the TaqMan Endogenous Control (Applied Biosystems) levels of each animal group. Graphs were plotted as arbitrary units (a.u.) based on log RQ values.

### Isolation of Hepatic Leukocytes

Mice were euthanized and slowly perfused with an intracardiac PBS flux. Livers were homogenized through a tissue strainer and intrahepatic leukocytes were obtained after a 20 min centrifugation (600 *g*) in a 35 and 70% bilayer Percoll gradient (Sigma). Viable cells were counted on a Newbauer’s chamber by trypan blue exclusion.

### Flow Cytometry

Cell suspensions were washed in ice-cold FACS buffer (PBS-2% FBS) and incubated with fluorochrome-labeled Abs for 20 min at 4°C. Cells were surface-stained with the following Abs properly combined: APC-eFluor780-anti-CD4, FITC-anti-CD8, APC-eFluor780-anti-F4/80, PE-anti-IL-1R, and APC-anti-IL-18R. Intracellular cytokines were detected after stimulating cells for 4 h with 30 ng/mL PMA and 500 ng/mL ionomycin (Sigma) in the presence of GolgiStop and GolgiPlug (BD Biosciences). Cells were then fixed and permeabilized with BD Cytofix/Cytoperm and Perm/Wash (BD Biosciences) according to the manufacturer’s instructions. Cells were incubated with: PerCp-Cy5.5-anti-IFN-γ, PE-anti-IL-17A, PeCy7-anti-IL-10, and APC-anti-IL-4, FITC-anti-TLR9 (Abcam). Also, goat anti-NLRP3 (Abcam) was used with Alexa-Fluor-633-anti-goat (Thermo-Fisher). No staining controls were included in each experiment in order to set the positive markers.

Oxidation-sensitive dye 10 µM H2DCFDA (Invitrogen) and 10 µM DAF-FM (Molecular Probes) were used to measure ROS and NO production, respectively (33). After washing, the samples were examined using a BD FACS Canto II flow cytometer (BD Biosciences), and then analyzed using the Flow Jo Software. Gate strategies are indicated in Figure S1 in Supplementary Material.

### Western Blot

Liver samples were lysed (1% Triton X-100, 0.5% sodium deoxicholate, 9% SDS, 1 mM sodium ortovanadate, and 10 μg PMSF in PBS), separated on a 10% SDS-PAGE and transferred to nitrocellulose membranes. After blocking, they were incubated with goat polyclonal anti-caspase-1-p20 (Santa Cruz) and rabbit polyclonal anti-NLRP3 (Abcam), detected with IRDyes and analyzed on an OdysseyCLx Imaging System (Li-cor). Protein loading was assessed with a polyclonal anti-actin Ab (Santa Cruz). Bands were quantified using Gel-Pro Analyzer software.

### Measurement of Cytokines

Tumor necrosis factor, IL-6, IL-10, and IFN-γ levels were determined by ELISA sandwich with antibody pairs purchased from eBioscience according to the manufacturer’s protocol in plasma. IL-1β and IL-18 were quantified with ELISA kits (eBioscience). The absorbance values were determined using a spectrophotometric plate reader (BIO-RAD, Model 680).

### Histological Studies of Liver Tissues

Liver tissue was fixed in 10% paraformaldehyde, embedded in paraffin, sectioned on slides, and stained with hematoxylin and eosin (H&E). Serial liver sections from different hepatic lobules were double blind analyzed and inflammatory infiltrate area was quantified vs. total area per field from five pictures randomly chosen from two mice of each group. Images were analyzed using Axiovision 4.8 (Zeiss) software.

### Fluorescence Microscopy

Hepatic leukocytes were put on a slide by the citospin technique, fixed in 4% paraformaldehyde, blocked with PBS-BSA 1%, and labeled with FITC-anti-F4/80, goat-anti-NLRP3 (Abcam), rabbit-anti-TLR9 (Abcam) were revealed with A633-anti-rabbit and A495-anti-goat (Thermo-Fisher) and visualized using an FV1000 confocal microscope (Olympus). Nuclei were stained with 2 µg/mL DNA-binding fluorochrome Hoechst 33258.

### Measurement of Transaminases and LDH Enzyme Activity

Blood samples were collected and the activity levels of aspartate aminotransferase (AST), alanine aminotransaminase (ALT), and lactate dehydrogenase (LDH) in plasma were quantified by Biocon Laboratory, Córdoba-Argentina.

### Statistical Analysis

Results are expressed as mean ± SEM. Comparisons among groups were performed using the parametric analysis of variance (one way or two-way ANOVA) followed by a multiple-comparison test (Bonferroni test) using Graph Pad Prism Inc., La Jolla, CA, USA. A *p*-value <0.05 was considered significant.

## Results

### *T. cruzi* Infection Triggers NLRP3 Inflammasome Response in the Liver and Increases the Number of NLRP3+ Hepatic Macrophages in WT Mice

Previous works have shown that NLRP3 inflammasome participate in the innate immune response to *T. cruzi* infection (28–30). However, it is unknown if in the liver immunity the NLRP3 inflammasome is triggered in the *T. cruzi-*Tulahuen strain. NLRP3 and ASC expression were analyzed in liver tissue of infected B6 WT by qPCR.

**Figure 1:**
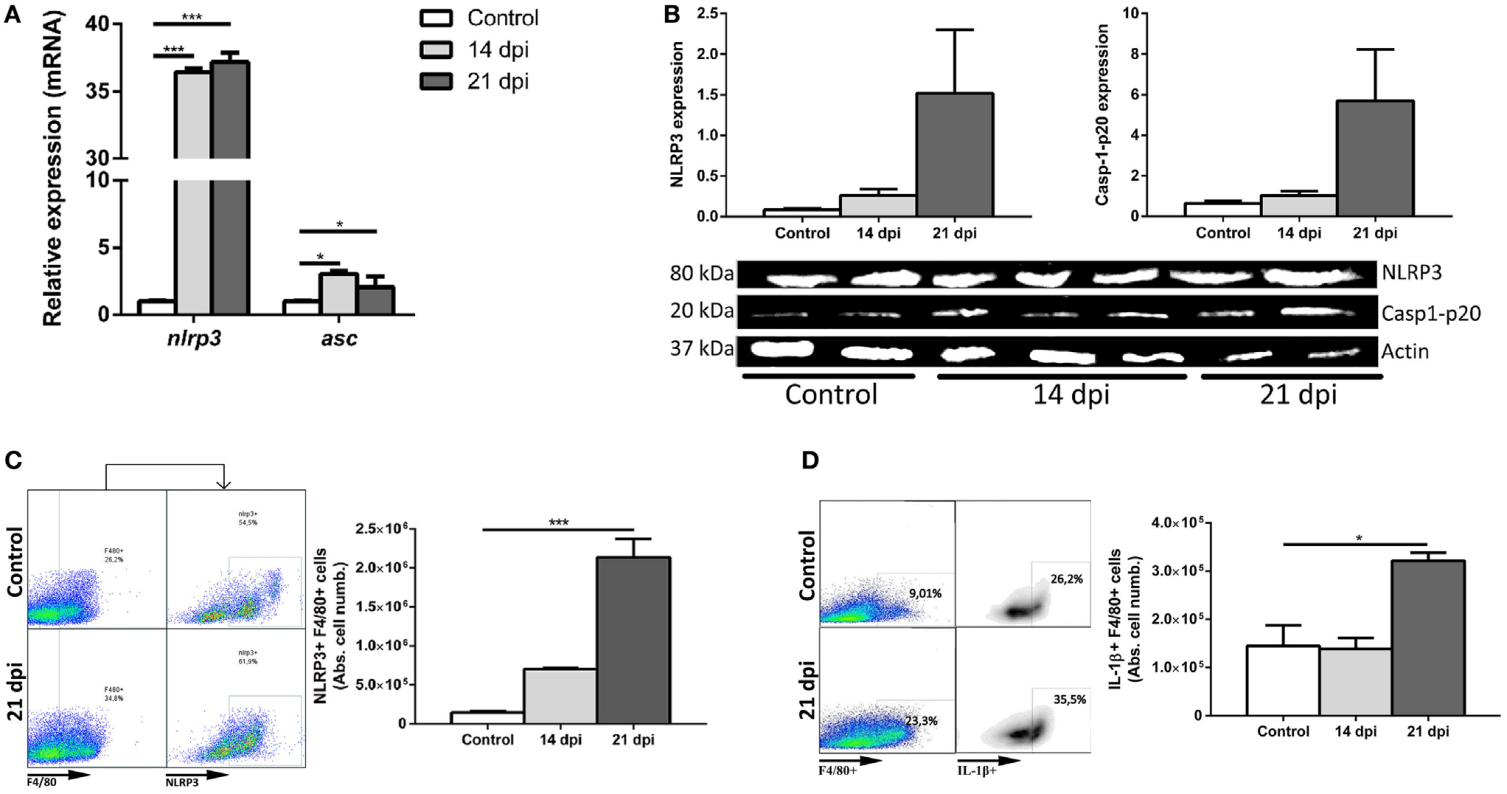
*Trypanosoma cruzi* triggers NLRP3 inflammasome response in the liver of wild type (WT) mice. Male WT mice were intraperitoneally inoculated with 10^3^ trypomastigotes-Tulahuen strain and livers were collected and perfused at 14 and 21 days post infection (dpi). **(A)** Hepatic transcript levels of *nlrp3* and *asc*. **(B)** Cropped lanes of protein expression of NLRP3 and caspase-1-p20 active subunit of liver samples assessed by Western blotting (uninfected controls, *n* = 2; 14 dpi, *n* = 3; 21 dpi, and *n* = 2). **(C)** Representative dot plots and absolute number of hepatic NLRP3-expressing F4/80+ cells. **(D)** Representative dot plots and absolute number of IL-1β-producing F4/80+ cells. Gate strategies (Figure S1A in Supplementary Material) were performed using anti-F4/80+ and anti-NLRP3 plus A633-anti-goat fluorochrome-labeled antibodies. Data are shown as mean ± SEM from one of three representative experiments; *n* = 2–6 mice per group. Statistical significance was evaluated by Two-way ANOVA followed by Bonferroni *post hoc* test. *p < 0.05; **p < 0.01; ***p < 0.001.

A strong increase of NLRP3 was found at 14 and 21 dpi in comparison with uninfected controls (Figure 1A). We also observed an increase in the protein levels of NLRP3 and caspase-1-p20 active enzymatic subunit by Western blot (Figure 1B).

Taking into account that during the acute infection with the Tulahuen strain, the liver of B6 mice displays cellular infiltrates with predominance of macrophages, we decided to analyze the induction of NLRP3 inflammasome and IL-1β within these cells. Hepatic macrophages (F4/80+ cells) are represented by Kupffer cells or resident macrophages plus inflammatory infiltrating macrophages/monocytes. Thus, we observed an increased absolute number of hepatic NLRP3+F4/80+ cells (Figure 1C). Additionally, we also demonstrated the presence of IL-1β in hepatic macrophages at 21 dpi by flow cytometry (Figure 1D). Analysis of the absolute cell number is fundamental due to the hepatomegaly induced during the acute *T. cruzi* infection.

### Infected Caspase-1/11-Deficient Mice Exhibit an Increased Number of Macrophages Associated With a Reduction of Hepatic CD8+ T Cells

**Figure 2:**
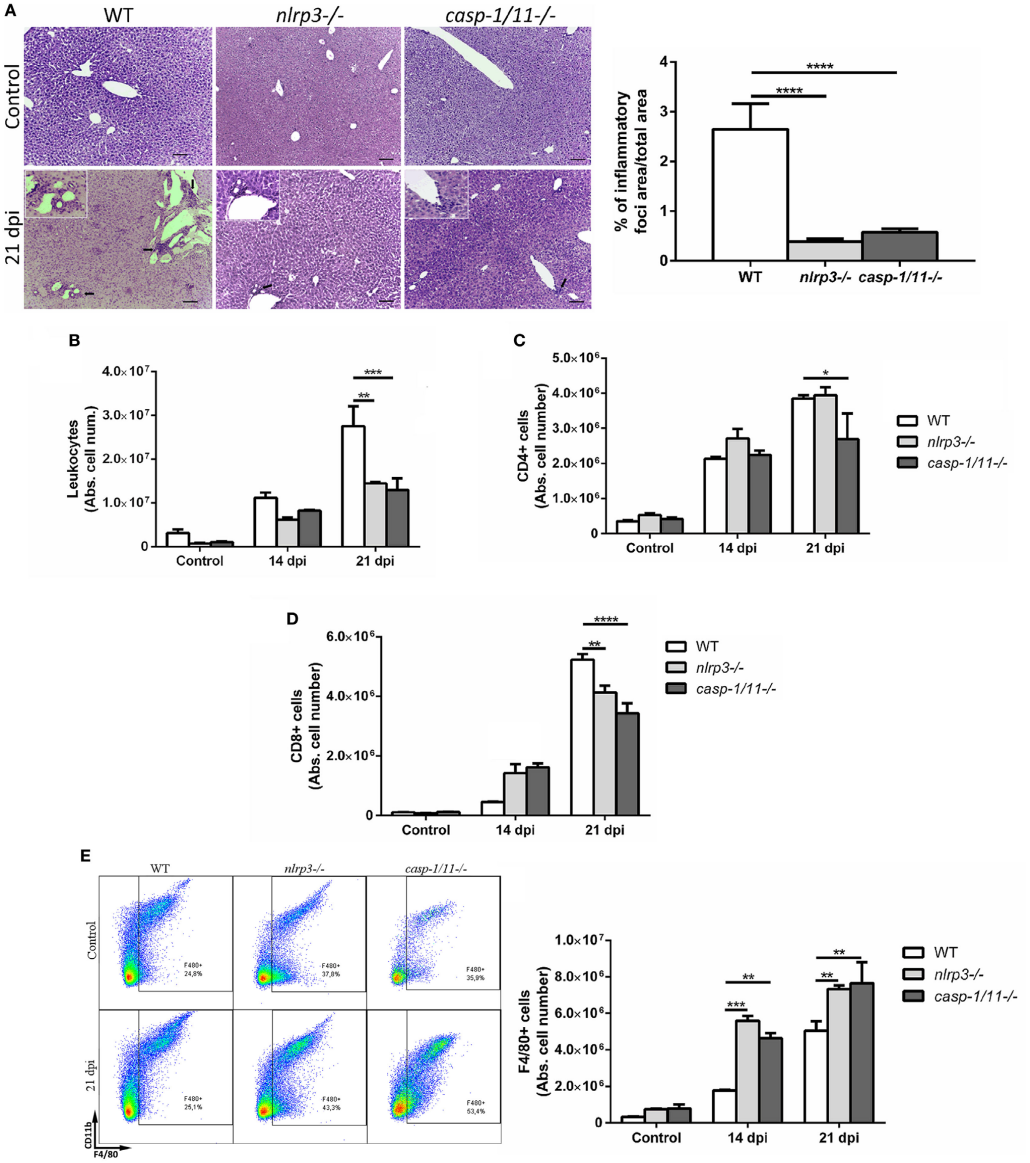
Infected caspase-1/11-deficient mice exhibit a marked reduction of hepatic CD8+ T cell number associated with increased number of macrophages. **(A)** Representative liver sections from uninfected control and *Trypanosoma cruzi* infected wild type (WT) and KO mice at 21 days post infection were stained with hematoxylin and eosin. Micrographs are shown at 100× and bar scales depict 50 µm. Insets are magnifications of inflammatory infiltrates. Bars depict percentages of inflammatory foci. **(B)** Absolute number of hepatic leukocytes from WT, *nlrp3−/−*, and *caspase-1/11−/−* mice determined by flow cytometry. **(C)** Absolute number of purified hepatic CD4+ and **(D)** CD8+ T cells. **(E)** Representative dot plots and absolute cell number of F4/80+CD11b+and F4/80+CD11b*−* leukocytes. Gate strategy is depicted in Figure S1 in Supplementary Material. Data are shown as mean ± SEM from one of three representative experiments; *n* = 3–6 mice per group. Statistical significance was evaluated by two-way ANOVA followed by Bonferroni *post hoc* test. *p < 0.05; **p < 0.01; ***p < 0.001.

**Figure 3:**
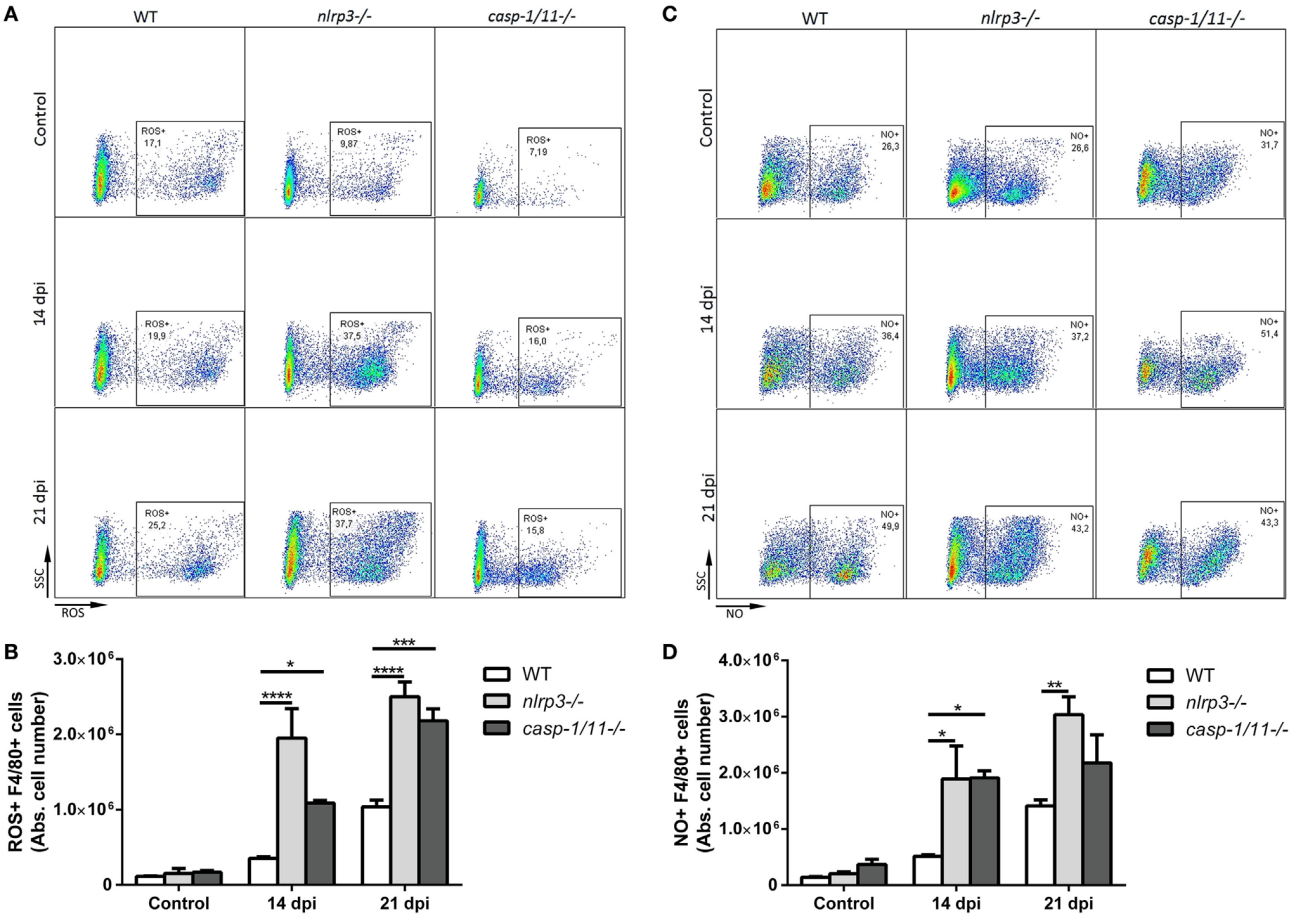
Hepatic macrophages from NLRP3- and caspase-1/11-deficient mice exhibit increased intracellular production of reactive oxygen species (ROS) and nitric oxide. Intrahepatic leukocytes from infected and uninfected controls from wild type (WT), *nlrp3−/−*, and *caspase-1/11−/−* mice were stimulated with PMA and ionomycin. ROS were detected within cells by labeling with the oxidation-sensitive dye H2DCFDA and NO was measured using DAF-FM as described in Section “Materials and Methods.” Then, cells were stained with anti-F4/80. **(A)** Representative dot plots and **(B)** absolute number of ROS+F4/80+ intrahepatic cells from WT, *nlrp3−/−*, and *caspase-1/11−/−* mice. **(C)** Representative dot plots and **(D)** absolute number of hepatic NO+F4/80+ cells from WT, *nlrp3−/−*, and *caspase-1/11−/−* mice measured by flow cytometry. Gate strategy is depicted in Figure S1 in Supplementary Material. Data are shown as mean ± SEM from one of three representative experiments; *n* = 3–6 mice per group. Statistical significance was evaluated by two-way ANOVA followed by Bonferroni *post hoc* test. *p < 0.05; **p < 0.01; ***p < 0.001.

In order to elucidate how NLRP3 and caspase-1/11 impact on innate and adaptive immune responses, we infected WT, *nlrp3−/−*, and *caspase-1/11−/−* mice and analyzed the hepatic leukocyte infiltration at 14 and 21 dpi. A reduced number of inflammatory foci and total infiltrating cells were found in both deficient strains compared with WT mice at 21 dpi (Figures 2A,B). When we analyzed the absolute number of hepatic T cell populations (Figures 2C,D), we found a significant decrease of CD8+ T cells in caspase-1/11*−*/*−* compared with those of WT mice at 21 dpi. Furthermore, we observed a higher increase in the number of F4/80+ cells in both deficient compared with those of WT mice (Figure 2E). Taking into account that TLR9 was strongly upregulated in the liver of infected WT mice and that this is linked to an exacerbated inflammatory response with hepatic damage (31), we first evaluated the intracellular expression of TLR9 and NLRP3 in hepatic macrophages of WT mice. We found a co-expression of both innate receptors in isolated macrophages of WT mice by confocal microscopy (Figure S2A in Supplementary Material). Additionally, we observed a higher number of TLR9+F4/80+ cells in infected *nlrp3−/−* compared with that of WT and *caspase-1/11−/−* mice as measured by flow cytometry (Figure S2B in Supplementary Material). Considering that macrophages may exert their microbicidal activity through production and release of oxidative stress mediators, we next examined the ability of the liver isolated macrophages of each mouse strain to produce ROS and NO intracellularly. As shown in Figure 3, we observed that the infection increased the recruitment of ROS- (Figures 3A,B) and NO-producing macrophages from all analyzed groups (Figures 3C,D).

### Infected NLRP3 and Caspase-1/11-Deficient Mice Differentially Modulate Helper and Cytotoxic T Lymphocyte Phenotypes During *T. cruzi* Infection

**Figure 4:**
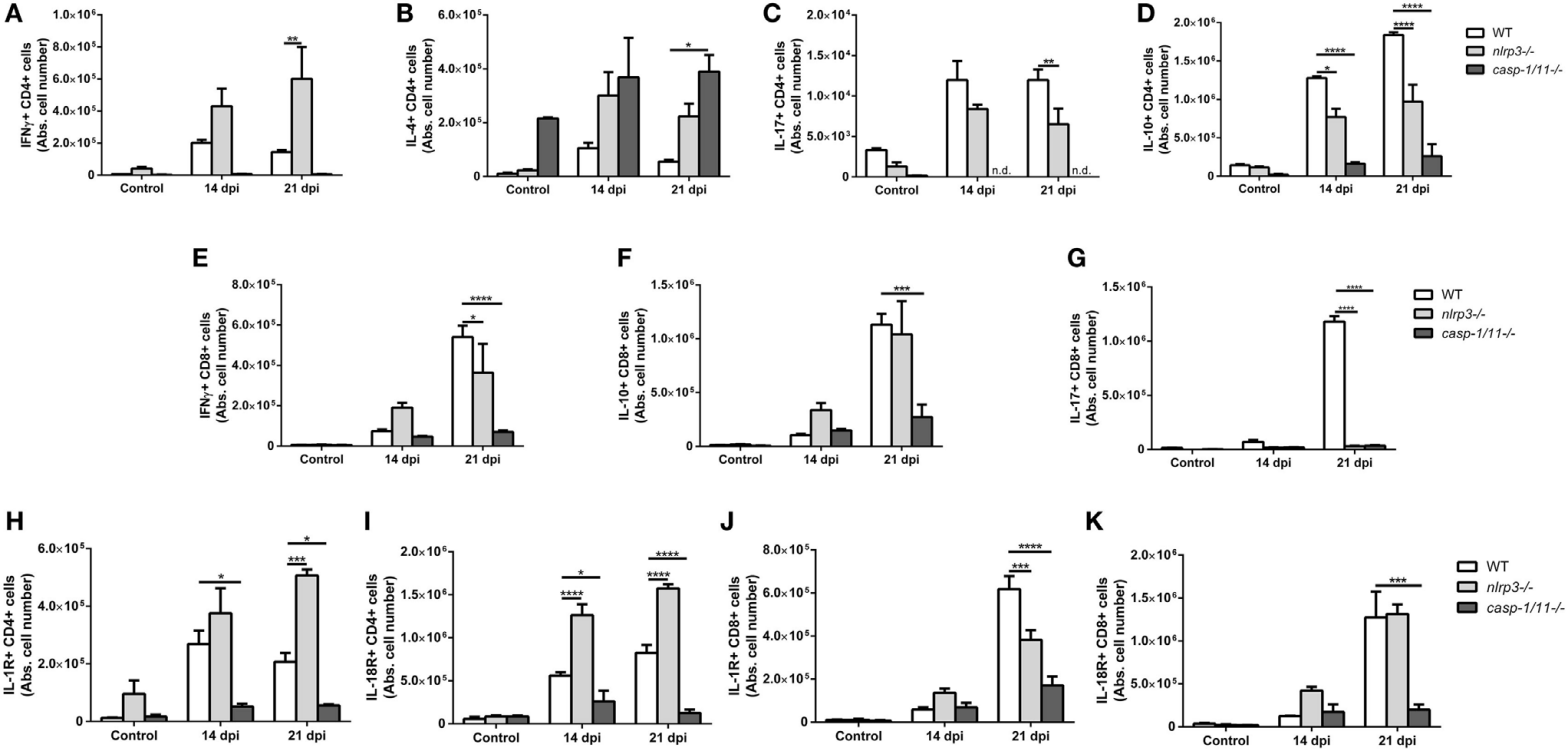
Infected NLRP3 and caspase-1/11-deficient mice differentially modulate T helper and T cytotoxic lymphocyte phenotypes during *Trypanosoma cruzi* infection. Intracellular cytokines were determined in hepatic leukocytes obtained at 14 and 21 days post infection by flow cytometry. Absolute number of hepatic **(A)** IFNγ+, **(B)** IL-4+, **(C)** IL-17+, and **(D)** IL-10+ in CD4+ T lymphocytes are shown. Absolute number of hepatic **(E)** IFNγ+, **(F)** IL-10+, and **(G)** IL-17+ in CD8+ T lymphocytes are shown. Absolute number of **(H)** IL-1R+ and **(I)** IL-18R+ on CD4+ T lymphocytes. Absolute number of hepatic **(J)** IL-1R+ and **(K)** IL-18R+ on CD8+ T lymphocytes. Data are shown as mean ± SEM from one of three representative experiments; *n* = 3–6 mice per group. Statistical significance was evaluated by two-way ANOVA followed by Bonferroni *post hoc* test. *p < 0.05; **p < 0.01; ***p < 0.001; ****p < 0.0001.

Besides innate immune cells, T cells of adaptive immunity play an important role in the protection against *T. cruzi* infection. Activated CD4+ and CD8+ T cells produce IFN-γ which further enhance the activation of phagocytic cells to promote parasite killing (23, 34). Consequently, we analyzed different subsets of liver CD4+ T lymphocytes elicited during *T. cruzi* infection and measured the characteristic intracellular cytokines for each subtype: IFN-γ+CD4+ (Figure 4A), IL-4+CD4+ (Figure 4B), IL-17+CD4+ (Figure 4C), and IL-10+CD4+ (Figure 4D). Interestingly, we found a mixed Th1/Th2/Th17 phenotype in *nlrp3−/−* similar to infected WT mice, whereas *caspase-1/11−/−* animals displayed a predominant Th2 phenotype at 21 dpi. Furthermore, WT and *nlrp3−/−* groups were also able to recruit IFN-γ+CD8+ (Figure 4E) and IL-10+CD8+ cells (Figure 4F), whereas *caspase-1/11−/−* animals exhibited scarce numbers of these CD8 T cell subsets. The only subset of CD8 T cell affected in both NLRP3 and caspase-1/11-deficient mice was IL-17+CD8+ (Figure 4G). Altogether, our results clearly demonstrate that the deficiency in *caspase-1/11−/−* worsens the induction of the cytotoxic response during the infection with this parasite.

Taking into account that inflammasomes are key signaling platforms to detect intracellular pathogens and release bioactive IL-1β and IL-18, which exert their biological effects through their specific receptors, we analyzed the expression of IL-1R and IL-18R on hepatic T lymphocytes. Thus, we found that *nlrp3−/−* animals had a higher number of IL-1R+CD4+ (Figure 4H) and IL-18R+CD4+ (Figure 4I) lymphocytes, whereas *caspase-1/11−/−* mice showed the lowest numbers of these cells when compared to infected WT mice. Additionally, the number of IL-1R+CD8+ (Figure 4J) and IL-18R+CD8+ (Figure 4K) dramatically decreased in *caspase-1/11−/−* animals compared to infected *nlrp3−/−* and WT group.

### Differential Systemic Levels of Cytokines in WT, *Nlrp3−/−*, and *Caspase-1/11−/−* Mice During *T. cruzi* Infection

**Figure 5:**
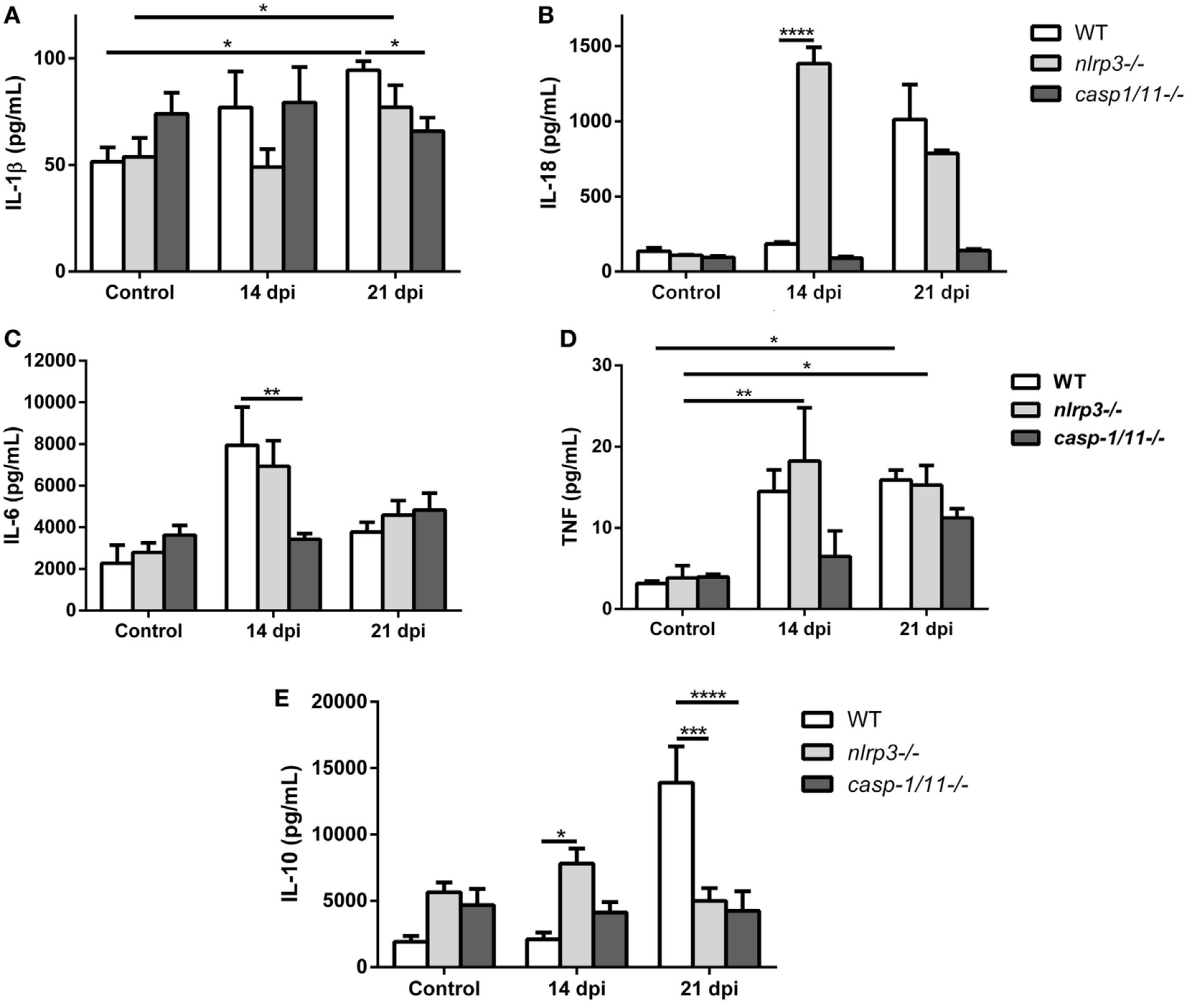
Differential systemic levels of cytokines in wild type (WT), *nlrp3−/−*, and *caspase-1/11−/−* mice during *Trypanosoma cruzi* infection. Plasma levels of the indicated cytokines were determined in uninfected and infected WT, *nlrp3−/−*, and *caspase-1/11−/−* at 14 and 21 days post infection. **(A)** IL-1β, **(B)** IL-18, **(C)** IL-6, **(D)** tumor necrosis factor, and **(E)** IL-10 were measured by ELISA Sandwich. Data are shown as mean ± SEM from one of three representative experiments; *n* = 3–6 mice per group. Statistical significance was evaluated by two-way ANOVA followed by Bonferroni *post hoc* test. *p < 0.05; **p < 0.01; ***p < 0.001; ****p < 0.0001.

Recognition of *T. cruzi* by the innate and adaptive immune cells trigger the production of cytokines, such as IL-1β, IL-18, IL-6, TNF, and IL-10 during the acute phase of infection which exert profound effects in the killing of parasites and in the modulation of inflammation. Thus, we found that the infection increased the plasma levels of IL-1β (Figure 5A) and IL-18 (Figure 5B) in WT and *nlrp3−/−* mice but not in *caspase-1/11*-deficient animals. Surprisingly, IL-18 concentration was strongly augmented in plasma from infected *nlrp3−/−* compared to WT mice at 14 dpi.

When we analyzed pro-inflammatory cytokines such as IL-6 in the plasma of both deficient mice, we noted that only *nlrp3−/−* mice produced an increase in this cytokine in a manner similar to WT mice at 14 dpi (Figure 5C). Additionally, high TNF concentrations were also detected in both WT and *nlrp3−/−* mice at 14 and 21 dpi, whereas in the *caspase-1/11−/−* group this cytokine was only increased at 21 dpi (Figure 5D).

Last, the anti-inflammatory cytokine IL-10 was strongly increased in WT mice at 21 dpi, whereas *nlrp3−/−* and *caspase-1/11−/−* animals did not show significant changes along the infection. It is worth noting that uninfected NLRP3 and caspase-1/11-deficient mice showed higher baseline levels of IL-10 than WT mice did (Figure 5E).

### Caspase-1/11 Signaling Is Required for Controlling *T. cruzi* Infection but Contributes to Liver Inflammation

**Figure 6:**
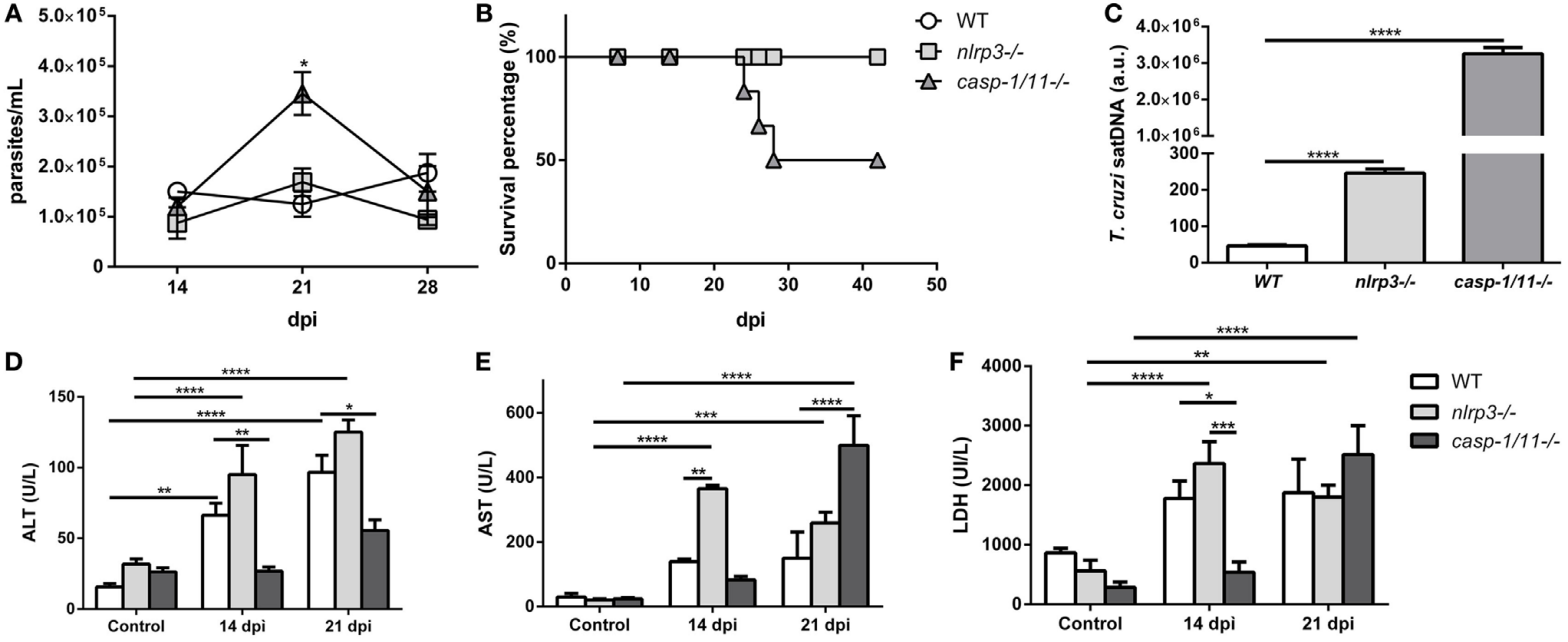
Caspase-1/11 signaling is required for controlling *Trypanosoma cruzi* infection but contributes to liver inflammation. Wild type (WT), *nlrp3−/−*, and *caspase-1/11−/−* mice were i.p. inoculated with 10^3^ trypomastigotes-Tulahuen strain. **(A)** Parasitemia levels were examined microscopically at the indicated time points. **(B)** Survival percentages from infected WT and *KO* mice up to 42 days post infection (dpi). **(C)** Hepatic parasitic load was determined by qPCR of satDNA of *T. cruzi* relative to TaqMan endogenous control and expressed as arbitrary units (a.u.) from infected WT, *nlrp3−/−*, and *caspase-1/11−/−* mice at 21 dpi. Activity levels of **(D)** ALT, **(E)** AST transaminases, and **(F)** LDH in plasma are obtained at 14 and 21 dpi. Data are shown as mean ± SEM from one of three representative experiments; *n* = 3–6 mice per group. Statistical significance was evaluated by two-way ANOVA followed by Bonferroni *post hoc* test. *p < 0.05; **p < 0.01; ***p < 0.001; ****p < 0.0001.

In order to evaluate the role of NLRP3 and caspase-1/11 in the control of the parasite infection *in vivo*, we analyzed the parasitemia and survival curves in the three mouse strains. These parameters were similar in WT and *nlrp3−/−* animals (Figure 6A). Remarkably, *caspase-1/11−/−* mice reached a parasitemia peak at 21 dpi with 50% of survival at 28 dpi compared to the *nlrp3−/−* and WT groups (Figure 6B). Surprisingly, even though *nlrp3−/−* mice did not show differences in parasitemia and survival, the parasitic load was higher in the liver of *nlrp3−/−* compared to WT animals. However, the highest parasite burden was detected in *caspase-1/11−/−* group at 21 dpi (Figure 6C).

We also measured plasma ALT (Figure 6D) and AST (Figure 6E) transaminases as well as LDH (Figure 6F) activities as biomarkers of tissue damage. We observed increased levels of these markers in both WT and *nlrp3−/−* mice at 14 and 21 dpi. It is worth noting that caspase-deficient mice showed a delayed increase in ALT and AST transaminases and LDH activities at 21 dpi likely due to the high parasitic load in the liver and other tissues.

## Discussion

In this study, we investigated the role of the inflammasome components in the protective and pathogenic adaptive immune responses induced in hepatic tissue, a key tissue during the acute infection with the Tulahuen strain. We demonstrated that parasite infection triggered NLRP3 inflammasome activation in the liver and increased the number of hepatic NLRP3+ and IL-1β+ macrophages in WT mice as observed in a viral hepatitis model (35). Liver resident macrophages together with those recruited by proinflammatory cytokines and chemokines are the major drivers of hepatic inflammation during acute and chronic *T. cruzi* infection (36, 37). In this study, livers of infected WT mice showed an increased number of inflammatory cell infiltrates compared to NLRP3 and caspase-1/11-deficient mice, suggesting that the inflammasome pathway is important for cell recruitment and hepatic inflammation (38). Furthermore, our results clearly show that NLRP3 inflammasome is dispensable for controlling parasitemia, host survival, and the onset of adaptive immune response. In this sense, other researchers focusing on heart tissue reported that inflammasome activation was partially dependent on NLRP3 and fully dependent on caspase-1 and/or ASC molecule to control cardiac inflammation, parasitism, and the survival of B6 mice infected with *T. cruzi-*Y strain (29). Indeed, host immune response against *T. cruzi* is an intricate phenomenon that may play different roles in dissimilar models depending on different parasite strains, the infected tissues and on the point in time at which the infection occurs among other factors. Strikingly, hepatic macrophages from nlrp3*−/−* mice showed an increased expression of TLR9 suggesting that this innate receptor is associated with NLRP3 signaling (Figure S2B in Supplementary Material). Supporting this idea, a recent report described how TLR9 negatively regulates the NLRP3–IL-1β pathway in an infection model (39). Moreover, the absence of NLRP3 could be inducing a positive feedback on TLR9 expression as has been proposed (40–42). Interestingly, the higher number of hepatic ROS+ and NO+ macrophages from infected *nlrp3−/−* and *caspase-1/11−/−* mice, prompted us to suggest that these anti-parasitic mechanisms would be relevant for an early control of infection (43). The trypanocidal activity attributed to NO is actually mediated by peroxynitrites (44) and IFN-γ and TNF pro-inflammatory cytokines may enhance the innate effector mechanisms against the pathogen (45–48). Furthermore, it was recently reported that, independently of caspase-1/11, TNF and ROS may activate macrophages to kill pathogens by recruiting lysosomes and acidifying pathogen-containing vacuoles (46). Interestingly, IL-17A cytokine also acts on macrophages enhancing the killing of intracellular parasites (12). Interestingly, a caspase-1 dependent and IL-1β and IL-18 independent pathway for NO production was described as an important effector mechanism played by NLRP3 to control *T. cruzi*-Y strain infection in vitro (28). Further investigation of this group demonstrated that lysosomal cathepsin B was required for NLRP3 activation, since its inhibition abrogates IL-1β release by macrophages (28). The molecular mechanisms of inflammasome activation that promote killing parasite inside macrophages infected by *T. cruzi*-Tulahuen strain *in vitro* remain unexplored and they are an interesting aspect of future studies.

On the other hand, IL-17A cytokine may also act on macrophages enhancing the killing of intracellular parasites (12). Interestingly, this cytokine was detected by us in hepatic CD4+ T cells from infected WT and NLRP3-deficient mice. The interplay between innate and adaptive immunity is very important in the progression and outcome of *T. cruzi* infection.

Our results showed that infected WT and *nlrp3−/−* animals displayed a mixed Th1, Th2, and Th17 phenotypes and exhibited similar systemic levels of IL-1β at 21 dpi. This proinflammatory cytokine promotes the activation and differentiation of lymphocytes, regulates the infiltration of inflammatory cells, induces chemotaxis and activation of other inflammatory factors, regulates the function of epithelial cells, and might produce tissue damage by mediating inflammation (49). In this study, we demonstrated that the induction of IFN-γ+CD4+ T cells (Th1) was predominant over IL-17+CD4+ T cells (Th17) in infected *nlrp3−/−* compared to WT mice. Conversely, the defect in caspase-1/11 signaling led to the lack of IL-1β induction and to a profound reduction of IFN-γ- and IL-17-producing T cells, which is in line with other reports (50, 51). Consequently, we observed a predominant Th2 phenotype on hepatic CD4+ lymphocytes from infected *caspase-1/11 KO* mice. Additionally, high levels of IL-18 were found in the plasma of infected NLRP3-deficient mice supporting the idea that IL-18 is a potent inducer of IFN-γ in Th1 (40). The bioactivity of this cytokine depends on its concentration, the level of its natural inhibitor, IL-18 binding protein, and the surface expression of IL-18R on the responding cells (52). In agreement with the above-described results, the high number of hepatic IL-18R+ and IL-1R+CD4+ T cells was detected in infected *nlrp3−/−* mice suggesting that different inflammasomes other than NLRP3 may be activated upon infection. As expected, the number of hepatic IL-18R- and IL-1R-expressing CD4+ T cells in *caspase-1/11−/−* mice did not suffer any change.

It is well known that production of IFN-γ by Th1 and Tc-1 cells is involved in the protection against *T. cruzi* (53). Moreover, cytotoxic T cells are essential for the control of infection in adaptive immunity since mice lacking CD8+ T cell compartment succumb to acute phase and display high systemic and tissue parasite loads (25). Besides, pre-clinical models of Chagas disease have demonstrated that antigen-specific IFNγ+CD8+ T cells are essential for reducing parasite burden, increasing survival, and decreasing pathology in both the acute and chronic phases of disease (54). In this study, we also observed the presence of IL-17-producing CD8+ T cells (Tc-17 subset) (55, 56) only in infected WT mice, suggesting the relevance of NLRP3 in the induction of this T cell sub-population. Interestingly, systemic IL-1β, IL-6, and TNF cytokines were associated with increased ALT and AST transaminases and LDH activities in infected WT and *nlrp3−/−* mice. The increase of AST may reflect the damage exerted by the parasite and inflammatory response in the different tissues as liver, spleen, heart, skeletal muscle, and kidneys, whereas ALT represent an hepatic injury biomarker. It is noteworthy that *caspase-1/11−/−* group exhibited a light increase of ALT though a strong increase in AST and LDH at 21 dpi probably due to the reduced infiltrated inflammatory foci in liver but increased and disseminated parasitemia. In line with our results, deficiency in caspase-1 resulted in high levels of parasitemia and increased number of amastigotes within macrophages isolated from caspase-1 KO mice and infected with *T. cruzi*-Y strain (28).

To sum up, our results provide the first evidence for the *in vivo* requirement of caspase-1/11 as an important player not only for mounting an appropriate type-1 adaptive immune response for the control of parasite burden in the liver, but also in promoting its inflammation and hence the progression of this parasite infection. We believe that these findings contribute to a better understanding of the inflammasomes in innate and adaptive responses as well as the mechanisms enabling parasite persistence within the host cells.

## Ethics Statement

All animal experiments were approved by and conducted in accordance with guidelines of the committee for Animal Care and Use of the Facultad de Ciencias Químicas, Universidad Nacional de Córdoba (Approval Number HCD 388/11) in strict accordance with the recommendation of the Guide to the Care and Use of Experimental Animals published by the Canadian Council on Animal Care (OLAW Assurance number A5802-01).

## Author Contributions

AP and SG conceived the experiments and wrote the manuscript. AP, PG, CD-L, AA, and LO collaborated with experiment performance. AP, PG, EC-S, RC, and SG analyzed the results. All authors reviewed the manuscript.

## Conflict of Interest Statement

The authors declare that the research was conducted in the absence of any commercial or financial relationships that could be construed as a potential conflict of interest.
