# Supplementary figures and images for: NLRP3 Inflammasome and Caspase-1/11 Pathway Orchestrate Different Outcomes in the Host Protection Against Trypanosoma cruzi Acute Infection
Source: Front Immunol. 2018 May 3;9:913. doi: 10.3389/fimmu.2018.00913 (PMC5944318; doi:10.3389/fimmu.2018.00913)

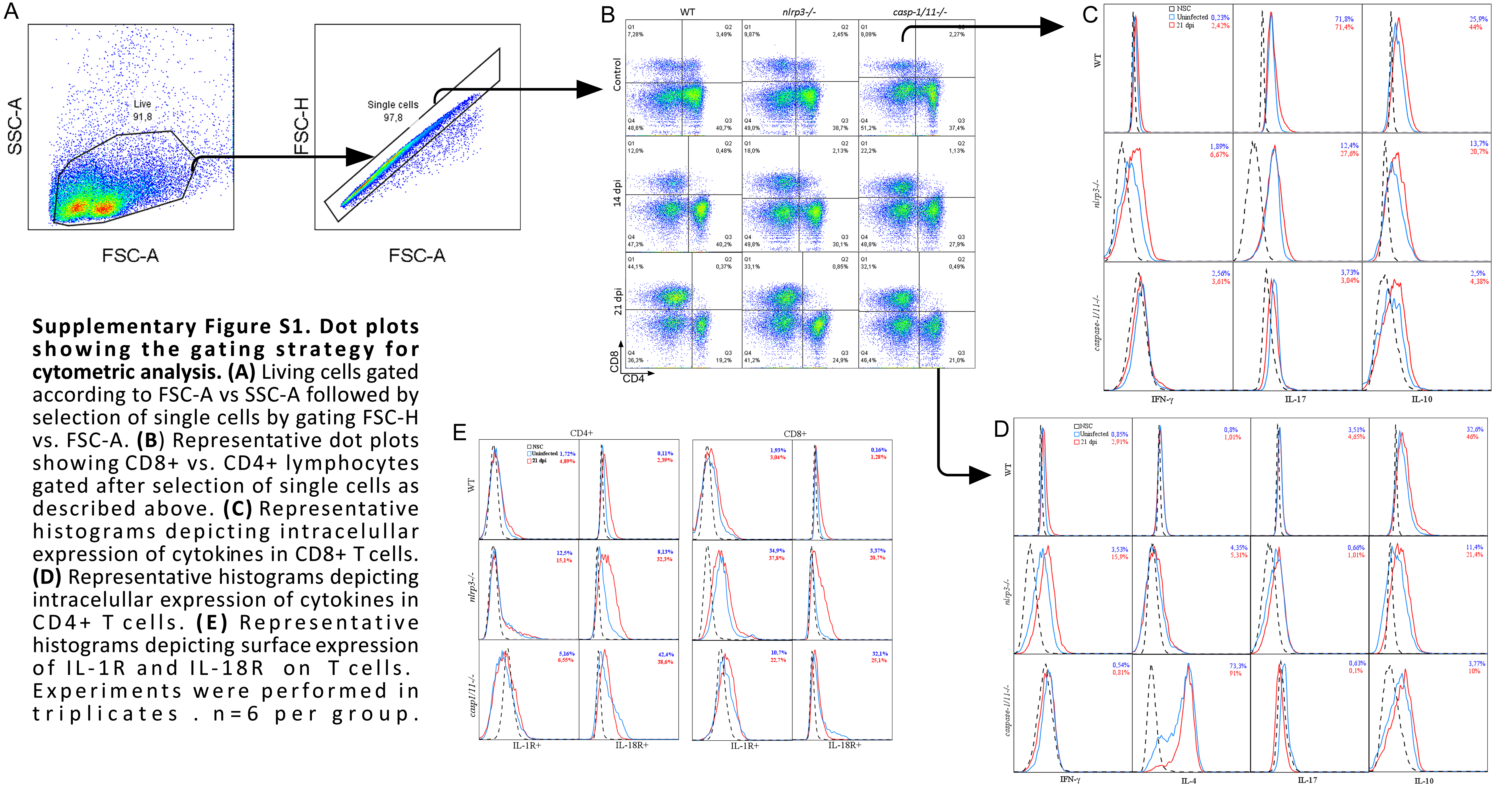

Supplement: Supplementary file 1 [file image_1.TIF]

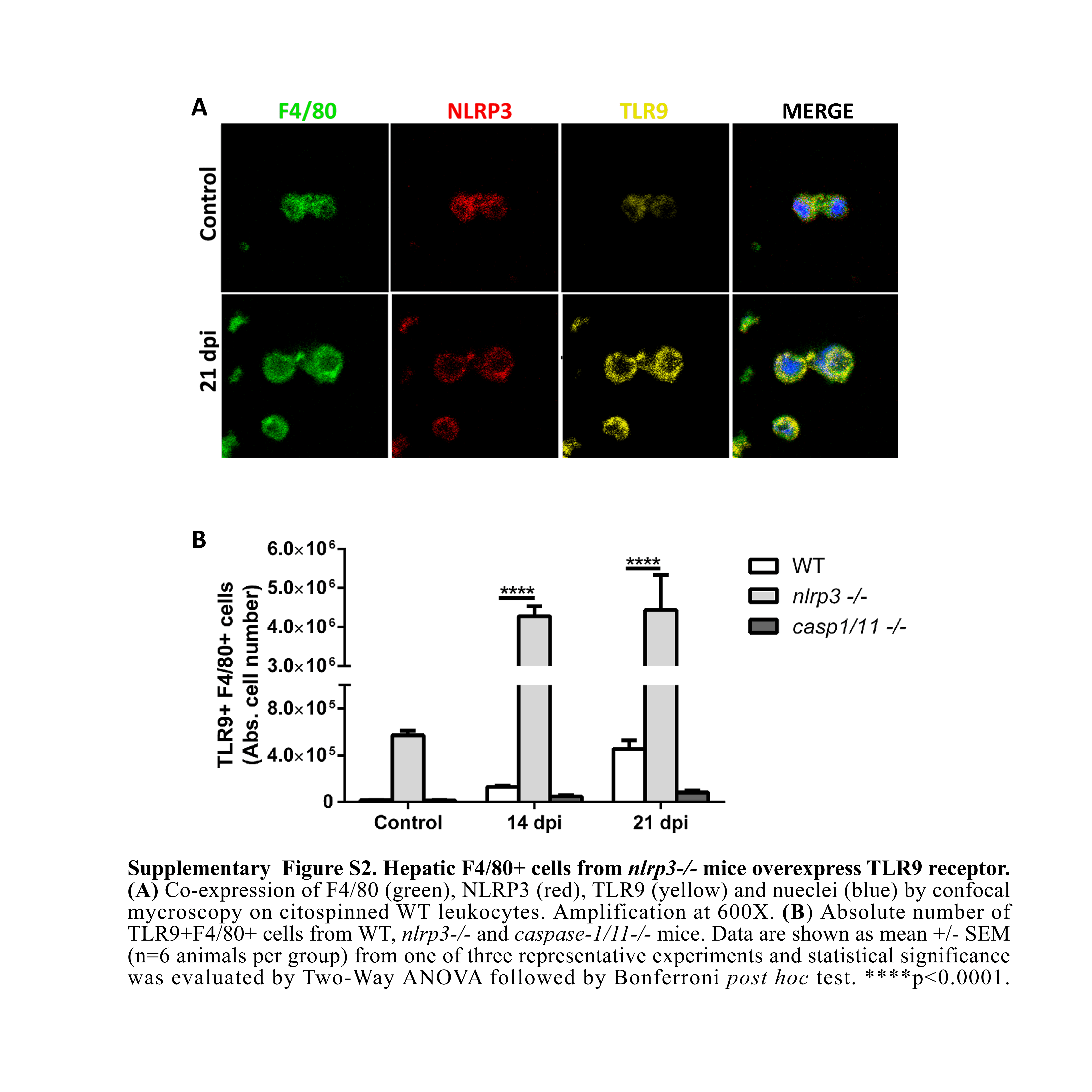

Supplement: Supplementary file 2 [file image_2.TIF]
